# Epigenetic Effects of Ethanol on the Liver and Gastrointestinal System

**DOI:** 10.35946/arcr.v35.1.06

**Published:** 2013

**Authors:** Shivendra D. Shukla, Robert W. Lim

**Affiliations:** **Shivendra D. Shukla, Ph.D.,***is Margaret Proctor Mulligan Professor, the Department of Medical Pharmacology & Physiology, School of Medicine, University of Missouri, Columbia, Missouri.*; **Robert W. Lim, Ph.D.,***is an associate professor in the Department of Medical Pharmacology & Physiology, School of Medicine, University of Missouri, Columbia, Missouri.*

**Keywords:** Ethanol, ethanol metabolism, alcohol consumption, epigenetics, epigenetic effects, epigenetic mechanisms, alcohol-induced epigenetic alterations, liver, gastrointestinal system, immune system, DNA methylation, histone acetylation, microRNAs (miRNAs), cell-signaling, oxidative stress, alcoholic liver disease, steatohepatitis, liver cancer, hepatic carcinoma

## Abstract

The widening web of epigenetic regulatory mechanisms also encompasses ethanol-induced changes in the gastrointestinal (GI)–hepatic system. In the past few years, increasing evidence has firmly established that alcohol modifies several epigenetic parameters in the GI tract and liver. The major pathways affected include DNA methylation, different site-specific modifications in histone proteins, and microRNAs. Ethanol metabolism, cell-signaling cascades, and oxidative stress have been implicated in these responses. Furthermore, ethanol-induced fatty liver (i.e., steatohepatitis) and progression of liver cancer (i.e., hepatic carcinoma) may be consequences of the altered epigenetics. Modification of gene and/or protein expression via epigenetic changes also may contribute to the cross-talk among the GI tract and the liver as well as to systemic changes involving other organs. Thus, epigenetic effects of ethanol may have a central role in the various pathophysiological responses induced by ethanol in multiple organs and mediated via the liver–GI axis.

Epigenetic modifications are emerging as important dynamic mechanisms contributing to both transient and sustained changes in gene expression. In some cases, epigenetic changes even can be inherited, although the mechanism for this remains elusive. Several types of epigenetic modifications have been studied in recent years. For example, several laboratories have actively examined modifications, of one end (i.e., the N-terminus) of the histone proteins around which the DNA is wrapped in the cell nucleus to form the chromatin. After their initial synthesis (i.e., after translation), histones can undergo a variety of modifications, such as acetylation, methylation, or phosphorylation, at different sites and under different conditions with diverse consequences. Another frequently studied type of epigenetic modification is the methylations of DNA at regions rich in cytosine and guanosine nucleotides (i.e., CpG islands), which has been found to affect, for example, cancer genes. Small RNA molecules called micro-RNAs (miRNAs) that cause inhibition of the first step of gene expression (i.e., transcription) or degradation of RNA also are considered to be master regulators involved in the modification of gene expression in abnormal conditions or disease states. Furthermore, all of these epigenetic mechanisms are influenced by foreign substances to which the body is exposed (i.e., xenobiotics) and environmental conditions.

The accumulation of all these findings has led to a dramatic shift from a genetic to an epigenetic basis in the conceptual thinking about the causes of disease. This also applies to the causes underlying ethanol-induced conditions, and new developments particularly have highlighted the importance of epigenetic mechanisms in mediating ethanol’s actions in the liver and gastrointestinal (GI) tract (see figure 1). These developments are the focus of this review.

## Alcohol-Induced Epigenetic Alterations in the Liver and GI Tract

### Histone Acetylation, Methylation, and Phosphorylation

Evidence for the ethanol-induced epigenetic modifications of histone H3 first was obtained by Park and colleagues (2003) who demonstrated H3 acetylation in primary cultures of rat liver cells (i.e., hepatocytes). Other researchers subsequently determined that ethanol altered methylation of histone H3 at two lysine residues (i.e., lys-4 and lys-9) (Pal-Bhadra et al. 2007) and that phosphorylation of histone H3 at two serine residues (i.e., ser-10 and ser-28) was increased in ethanol-exposed hepatocytes (Lee and Shukla 2007). Additional studies have established that these changes occur not only in cultured hepatocytes but also in vivo in the liver and other organs (see Kim and Shukla 2006; Shukla and Aroor 2006) as well as in other liver cell types (e.g., hepatic stellate cells) (Kim and Shukla 2005). Alcohols other than ethanol that can be found as contaminants in adulterated alcoholic drinks also can modify histones (Choudhury et al. 2008). Finally, by interfering with single-carbon metabolism, ethanol may potentiate the epigenetic effects of toxins released by certain bacteria in the GI tract (i.e., lipopolysaccharide or endotoxin). These toxins promote methylation of histone H3 at lys-4 (Ara et al. 2008), which could in turn contribute to the progression of alcoholic liver disease (ALD).

The histone proteins form larger complexes called nucleosomes around which the DNA is wound in the cell nucleus. Modifications at different sites in histone H3 (e.g., lys-4, lys-9, ser-10, ser-28, etc.) may occur on nucleosomes located in the same or different domains of the chromatin (James et al. 2012). These site-specific modifications, in turn, will be associated with changes in the expression of different genes with diverse effects. Thus, ethanol can influence an intricate network of epigenetic modifications.

It should be noted that although the observed global ethanol-induced changes in histone modifications suggest that they would result in large-scale, perhaps genome-wide, alterations in gene expression, epigenetic changes also can be limited to selected subsets of genes, depending to some degree on the method and mode of ethanol administration. Indeed, a gene-specific increase in H3K9 acetylation has been observed in rat liver in response to chronic ethanol feeding even in the absence of obvious global changes in histone acetylation (Park et al. 2012). Ethanol-induced histone modification is associated with altered expression of several genes, including those encoding the ethanol-metabolizing enzyme alcohol dehydrogenase (ADH), the cancer-promoting gene (i.e., oncogene) *c-jun*, and the gene encoding a protein called plasminogen activator inhibitor 1 (PAI-1), which is involved in the dissolution of blood clots and in various diseases (e.g., fibrosis and certain types of cancer) (see table 1).

### Changes in miRNAs

miRNAs are RNA molecules that do not serve as templates for protein production but have regulatory functions (for more information on miRNAs, see the article by Balamaran et al., pp. 18–24). To date, hundreds of miRNAs have been identified (Miranda et al. 2010) whose expression may be altered by various stimuli and as a result of changes in internal or environmental conditions. For example, chronic ethanol feeding results in up- or down-regulation of 1 percent or more of known miRNAs in the liver of mice (Dolganiuc et al. 2009) and rats (Dippold et al. 2013). Among those that were upregulated in rat liver by ethanol exposure were miR-34a, miR-103, miR-107, and miR-122 (Dippold et al. 2013), which have been implicated in the regulation of lipid metabolism (Esau et al. 2006; Lee et al. 2010), iron (Castoldi et al. 2011), and maintenance of glucose levels (i.e., glucose homeostasis) (Trajkovski et al. 2011). Conversely, the levels of miR-200b and miR-19b were downregulated under the same experimental conditions (Dippold et al. 2013). Similar results were observed in mice, where chronic ethanol feeding with a liquid Lieber-DeCarli diet led to upregulation of miR-705 and miR-1224 and downregulation of miR-182, miR-183, and miR-199a-3p in the liver. However, the biological targets of these miRNAs in the context of alcohol consumption still need to be determined (Dolganiuc et al. 2009; see table 1).

Ethanol exposure also influences miRNA expression in response to other changes in the organism. For example, the levels of a miRNA called miR-21 normally increase after a part of the animal’s liver is removed (i.e., after partial hepatectomy), which had been thought to contribute to the regeneration of the liver. Ethanol enhances this increase in miR-21 but paradoxically interferes with the regenerative process (Dippold et al. 2012). The significance of the miR-21 increase therefore remains to be elucidated.

Chronic ethanol feeding of mice and exposure of mouse hepatocytes to ethanol in vitro also induces miR-217 (Yin et al. 2012), which has been proposed to be linked to excess fat accumulation in the liver. Interestingly, this effect on fat metabolism seems to be correlated with reduced expression of an enzyme involved in histone modification (i.e., the class IV histone deacetylase [HDAC], SIRT-1). SIRT-1 is a molecular target not only of miR-217 but also of miR-34a (Lee et al. 2010) which, as indicated above, also is upregulated by ethanol (Dippold et al. 2013). Likewise, expression of another miRNA, miR-101, can downregulate the level of another enzyme involved in histone modification called histone methyltransferase Ezh2 (Cao et al. 2010). Although it is not known if miR-101 expression is affected by ethanol, these studies point to the intriguing possibility that change, in miRNA levels also could indirectly affect other epigenetic changes such as histone acetylation and methylation.

Changes in miRNA levels in response to ethanol are not limited to the hepatocytes but also affect other types of cells found in the liver and GI tract. For example, ethanol feeding leads to up-regulation of miR-20 and miR-203 as well as down-regulation of miR-135 and miR-199 in liver sinusoidal endothelial cells (Yeligar et al. 2009), and increases the levels of miR-132 and miR-155 in Kupffer cells (Bala et al. 2011). In addition, elevated levels of miR-212 have been detected in intestinal epithelial cells of patients with ALD (Tang et al. 2008). These changes in miRNA levels are correlated with altered expression of certain proteins in these cells, including increased expression of endothelin-1 (ET-1) and ET-1 receptor (ET-BR) in endothelial cells (Yeligar et al. 2009), increased expression of the proinflammatory cytokine tumor necrosis factor-α (TNF α) in Kupffer cells (Bala et al 2011), and reduced expression of a protein called zonula occludens 1 (ZO1), which helps ensure the tight connection between intestinal epithelial cells (Tang et al. 2008). As will be discussed later in this article, these changes in turn may contribute to the cross-talk between the liver and the GI and immune systems that ultimately may be responsible for the development of ALD.

**Figure 1:**
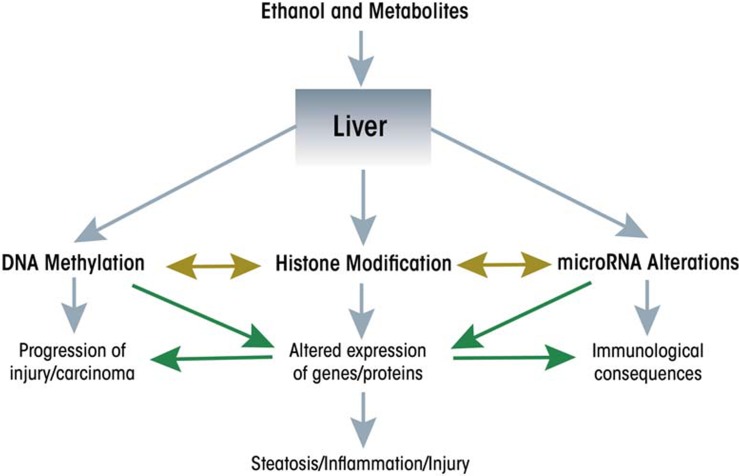
Ethanol and its metabolites modify epigenetic pathways in the liver.

Although changes in miRNA levels can affect expression of enzymes involved in other epigenetic modifications, it is equally clear that expression of miRNAs themselves can be subject to regulation by histone modifications and/or DNA methylation at the DNA regions that regulate miRNA expression (i.e., at their promoters). For example, the ethanol-induced expression of miR-155 seems to be regulated by the recruitment of a regulatory protein called nuclear factor κB (NFκB) to the miR-155 promoter (Bala et al. 2011), presumably accompanied by epigenetic changes associated with gene activation. In other studies, removal of methyl groups from (i.e., demethylation of) cytosine nucleotides at the promoters of miR-29a and miR-1256 correlated with upregulation of these miRNAs in prostate cancer cells (Li et al. 2012). Although it is not yet known whether miRNAs regulated by ethanol also may be regulated by DNA methylation, these studies clearly point to the intriguing possibility of cross-talk among molecular components involved in different types of epigenetic modifications (see figure 1).

### Changes in DNA Methylation Patterns

Ethanol also can alter the methylation patterns of DNA in liver, thereby influencing gene expression. For example, genes encoding enzymes involved in ethanol metabolism (e.g., ADH) are regulated by DNA methylation (Dannenberg et al. 2006). It therefore is likely that reduced levels of DNA methylation (i.e., hypomethylation) in response to ethanol will modulate the transcription of these genes. This effect is particularly relevant in patients with late-stage ALD, where ethanol is involved in the promotion of hepatic carcinoma. Like changes in miRNA expression, alcohol-induced changes in DNA methylation also have been observed in organs other than the liver. For example, chronic ethanol feeding in rats affects methylation of genes regulating absorption of the vitamin folate in the intestine (Wani et al. 2012). Folate is an important cofactor in single-carbon metabolism; therefore, its deficiency in turn could affect methylation reactions in various other organs, including the liver.

Kutay and colleagues (2012) found that ethanol affects methylation patterns by reducing the levels and activity of key DNA methylation enzymes, DNA methyl transferase (DNMT) 1 and 3b, without altering their mRNA levels. However, chronic ethanol feeding did not reveal any detectable methylation at the CpG islands in the promoters of several genes examined in liver (e.g., genes called *Agpat 9, Lepr*, and *Ppar*α), suggesting that promoter methylation may not be involved in regulating the expression of these genes. Instead, transcriptional activation or chromatin modification may be the predominant mechanism involved in ethanol-induced gene expression. This possibility has yet to be confirmed in additional studies, including studies in human liver.

Several observations suggest that changes in DNA methylation induced by diet, folate deficiency, or alcohol exposure may represent important epigenetic mechanisms. For example, chronic exposure to ethanol has been shown to produce DNA hypomethylation throughout the genome in the colonic mucosa in rats, and this hypomethylation may constitute a pathway by which carcinogenesis is enhanced (Choi et al. 1999). Other studies have focused on the role of a compound known as S-adenosylmethionine (SAMe), which acts as a methyl donor, in liver injury. Ethanol-induced alterations in SAMe levels can affect the methylation of histones or DNA, which in turn can modify gene expression, thereby contributing to liver injury (Lu and Mato 2012).

## Role of Ethanol Metabolism and Oxidative Stress in Ethanol-Related Epigenetic Mechanisms

The actions of ethanol in the liver are complex because it is metabolized via both oxidative and nonoxidative pathways that result in the generation of several metabolites, such as acetalde-hyde and acetate. Interestingly, both of these metabolites, as well as ethanol itself, increase histone H3 acetylation. This observation is supported by studies investigating the effects of inhibitors of ADH (i.e., 4-methyl pyrazole) and of another alcohol-metabolizing enzyme called aldehydyde dehydrogenase (i.e., methyl cynamide). These inhibitors prevented acetaldehyde and acetate formation and also reduced ethanol-induced increases in histone acetylation (Park et al. 2003), suggesting that ethanol metabolism has a role in this effect. Other findings suggest that ethanol-derived acetate may increase histone acetylation by increasing the available levels of acetyl groups for these reactions. Thus, studies in a cultured macrophage cell line found that downregulation of an enzyme that converts acetate into acetyl CoA, which then is used for histone acetylation, ameliorates the acetate effect on histone modification (Kendrick et al. 2010). However, the significance of this observation in vivo is unclear because the changes in acetyl-CoA levels following alcohol consumption are rather modest and transient.

Another important consequence of ethanol metabolism in the liver is the production of reactive oxygen species (ROS), leading to oxidative stress. ROS have been shown to play a role in ethanol-induced histone acetylation. Antioxidants that selectively interfere with different steps of ROS production affect this response. For example, general antioxidants (e.g., resveratrol or quercetin) inhibit histone acetylation. Conversely, inhibitors of certain enzyme complexes that are involved in ROS productions, such as rotenone (which inhibits mitochondrial complex 1) and antimycin (which inhibits mitochondrial complex 3) increase histone acetylation (Choudhury et al. 2010). These observations are consistent with the view that ROS contribute to the epigenetic effects of alcohol consumption.

## Role of Cell-Signaling Pathways in Ethanol-Related Epigenetic Mechanisms

The cellular actions of ethanol, including its epigenetic effects, are mediated via several signaling pathways (Mandarekar and Szabo 2009). One of these involves several enzymes called mitogen-activated protein (MAP) kinases (MAPKs) and therefore is known as the MAP kinase cascade. There are several different MAP kinase pathways that involve different MAPKs and which differentially affect ethanol-induced epigenetic modifications. For example, histone H3 phosphorylation is dependent on p38 MAPK (Lee and Shukla 2007), whereas histone H3 acetylation is regulated by a MAP kinase cascade involving MAPKs called ERK1/2 and JNK (Park et al. 2005). Even more intriguing is the finding that acetate-induced acetylation of histone H3 is MAPK independent (Park et al. 2005; Aroor et al. 2010). Thus, the involvement of different signaling pathways likely adds another level of regulatory control on histone modifications by ethanol and its metabolites (Shukla et al. 2013). These remarkable differences in signaling pathways utilized by ethanol and acetate may underlie the different modes of histone modifications and consequences of ethanol and its metabolites. This issue remains to be addressed in future studies.

## Role of Epigenetic Mechanisms in Ethanol-Induced Steatosis, Steatohepatitis, and Carcinoma

Excessive alcohol consumption can lead to a range of liver disorders, including fatty liver (i.e., steatosis), steatosis accompanied by inflammation of the liver (i.e., steatohepatitis), and progressing in some cases to liver cancer (i.e., carcinoma). Histone modifications, DNA methylation, and miRNA expression may all play roles in ethanol-related steatosis and inflammatory responses. For example, ethanol affects the activity of enzymes called histone acetyl transferases (HATs) that mediate histone acetylation. One of these ethanol-regulated HATs is called GCN5 (Choudhury et al. 2011); it modulates the expression of a protein called PGC1β, which is involved in fat metabolism in the liver (Kelly et al. 2009). Furthermore, chronic intragastric ethanol feeding of rats leads to an increase in the levels of another HAT called p300 in the cell nuclei at peak blood alcohol level, which is correlated with increased acetylation of H3-lys-9 (Bardag-Gorce et al. 2007).

Another type of histone-modifying enzyme are the HDACs. Chronic feeding of mice with an ethanol liquid diet downregulates the activity of the HDAC SIRT-1 and increases the expression of lipin-1, an important regulator of lipid synthesis in the liver (Yin et al. 2012) In contrast, other studies indicated that the transcription levels of SIRT-1 and PGC1β—another regulatory protein involved in lipid metabolism—are increased by chronic intragastric ethanol feeding in rats (Oliva et al. 2008). Recent studies also have shown that liver-specific knockout of the gene encoding HDAC3 in mice leads to severe hepatic steatosis and increased expression of lipogenic genes, although whether HDAC3 expression or function is altered by ethanol has yet to be elucidated (Sun et al. 2011). Increasing evidence thus suggest that both HATs and HDACs are likely to play a role in ethanol-induced liver injury (see Kirpich et al. 2012; Park et al. 2005; Pochareddy et al. 2012; Shepard et al. 2008; Yin et al. 2012). In addition to changes in lipid metabolism two molecules involved in inflammatory reactions (i.e., interleukin [IL] 8 and PAI-1) also are influenced by ethanol-induced histone modifications. Finally, ethanol-induced DNA hypomethylation has been implicated in the development of steatosis (Kutay et al. 2012) as well as hepatic carcinoma, an end consequence of ALD (Lambert et al. 2011).

miRNAs also mediate some of ethanol’s effects in causing liver disorders. For example, the down regulation of SIRT-1 in mice in response to ethanol feeding described above appears to be mediated by miR217 (Yin et al. 2012). A high-content screening of 327 human miRNAs identified 11 that when over-expressed in human hepatocytes lead to either increased or decreased intra-cellular lipid droplets, with miR-181d being the most efficacious inhibitor of lipid droplet formation (Whittaker et al. 2010). As discussed above, the immunological responses of liver macrophages are thought to involve miR-155 (Bala et al. 2012). Moreover, several miRNAs have been postulated to play a role in ethanol-induced intestinal defects (Tang et al. 2008) which could also indirectly exacerbate liver injury (see further discussion below).

## Time Dependence and Persistence of Alcohol-Induced Epigenetic Changes

**Figure 2:**
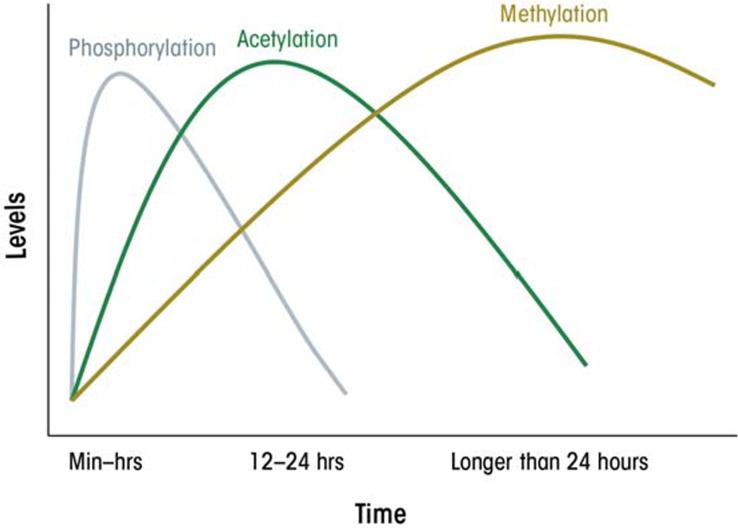
A schematic presentation of the kinetics of histone modifications by ethanol in liver.

Interestingly, the various epigenetic modifications observed in cultured hepatocytes in response to ethanol follow different time courses. For example, phosphorylation of H3 starts before acetylation and methylation of this histone (see figure 2). Furthermore, although the global changes more, although the global changes in histone modifications appear to be transient, with the effect peaking at about 24 hours following initial ethanol exposure, it is likely that these changes may trigger secondary changes in gene expression (including those of miRNA) or DNA modification that are much longer lasting. To date, little is known about the time course and sustainability of these other epigenetic modifications. It also is possible that even when the overall global changes in histone modification have subsided, some of the secondary changes may persist in nucleosomes associated with specific genes and may continue to influence expression of these genes.

The responses to ethanol consumption in vivo also have not yet been fully elucidated. Chronic ethanol treatment definitely results in abundant epigenetic changes months after the ethanol feeding began. How long these changes remain after withdrawal of alcohol has not been carefully evaluated with respect to the GI tract and liver. Studies in other organ systems, however, suggest that some of these changes could indeed persist for a long time. For example, prenatal exposure of rat fetuses to ethanol resulted in the development of hepatic insulin resistance in the off-spring 3 months after birth, which was correlated with an increase in HDAC activity and decrease in HAT activity in the liver (Yao and Nyomba 2008). Furthermore, exposure of males to ethanol was correlated with hypomethylation of normally hypermethylated regions in the DNA of the sperm corresponding to various paternally imprinted genes (Ouko et al. 2009). Epigenetic changes in these imprinted genes could be transmitted to the progeny following fertilization and thus affect the development and perhaps physiological functions of different organs, including the liver. Epigenetic effects of alcohol thus might even be able to exert long-lasting transgenerational effects in the offspring.

## Relationship to the Immune System

Evidence gathered in the past decade has clearly shown that ethanol alters several immunological parameters. One important participant in ethanol’s actions is a group of regulatory molecules called macrophage toll-like receptors (TLRs), particularly TLR 4. Ethanol’s effects on TLRs likely are mediated via miRNAs because, as mentioned earlier, ethanol increases the levels of several of these noncoding RNAs. Other studies have shown that ethanol influences the activities of different classes of TLR-regulated genes through distinct epigenetic histone modifications (Foster et al. 2007). Specifically, several pro-inflammatory genes are selectively deacetylated during the development of immune tolerance and are no longer inducible in the tolerant macrophages. It is tempting to speculate that by affecting histone modifications, ethanol could interfere with the development of tolerance and thus promote a chronic inflammatory state. Consistent with this idea, exposure of cultured macrophages to ethanol or ethanol metabolites resulted in increased production of TNF- (Shen et al. 2009), although whether this involves increased histone modification at the TNF- promoter remains to be established. In addition to the involvement of Kupffer cells, it is likely that interactions between activated hepatic stellate cells and hepatocytes also contribute to a pro-inflammatory environment by increasing the production of cytokines. This cross-talk between stellate cells and hepatocytes appears to be inhibited by deacetylase inhibitors, such as trichostatin (Coulouarn et al. 2012).

It should be pointed out that ethanol’s effects on the immune system likely are rather complex. In contrast to the enhanced inflammatory response seen during steatohepatitis following chronic ethanol administration, acute exposure to ethanol in vivo suppresses various inflammatory responses (e.g., leukocyte recruitment and endothelial cell activation) (Saeed et al. 2004). It is not completely clear if this anti-inflammatory effect is related to epigenetic changes; however, other studies have shown that treatment with HDAC inhibitors likewise inhibits the migration of macrophages in response to an inflammation-inducing stimulus (i.e., exposure to lipopolysaccharide) (Maa et al. 2010). Thus, it appears that ethanol may exert potent effects on the immune system, which likely are related to its epigenetic action, and that chronic and acute ethanol treatment could elicit different outcomes (Shukla et al. 2013).

## Cross-Organ Talk Between the Liver and GI Tract

The nutrients and xenobiotics taken up orally pass through the intestinal system and then to the liver, the major metabolic organ in the body. Ethanol can alter the permeability of the intestine, a condition known as leaky gut. This alcohol-induced gut leakiness is an important factor in ALD because it allows endotoxin to enter the circulation and initiate liver damage (Keshavarzian et al. 2009). The alcohol-induced gut leakiness may in part be caused by epigenetic changes to genes coding for proteins involved in joining epithelial cells to each other (i.e., epithelial cell junction proteins) (Tang et al. 2008). For example, alcohol induced overexpression of miR-212 and downregulated expression of the ZO1 protein. A decrease in ZO1 disrupts intestinal permeability and integrity, resulting in gut leakiness (Tang et al. 2008).

**Figure 3:**
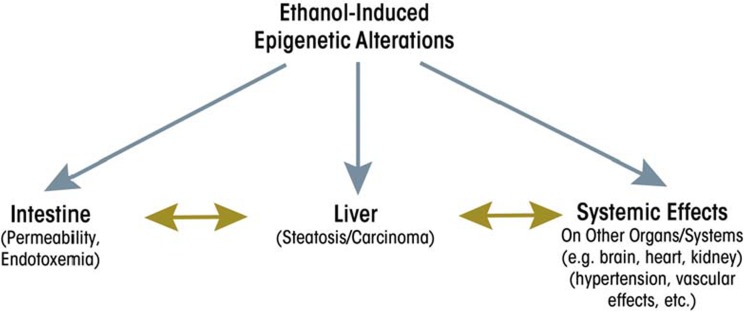
Ethanol-induced epigenetic alterations and cross-organ talk through the gastrointestinal–liver axis.

The response of the liver to ethanol and endotoxin is a complex process involving macrophage-like Kupffer cells, hepatocytes, and stellate cells. Alcohol’s effects on the activities of these cells may lead to liver injury and ultimately carcinoma. Ethanol causes epigenetic alterations in these cells that could result in changes in expression of genes associated with modified histones, including genes coding for various cytokines. Increases in the expression of these cytokines may occur in the liver, resulting in increased cytokine levels that then are circulated through the blood to other organs (e.g., heart or kidney) and in turn affect the functions of these organs. Thus, alcohol-induced epigenetic effects in the liver eventually may influence the cross-talk among these organs (see figure 3). This will be a fruitful topic for future studies to fully comprehend the role of ethanol-induced epigenetic alterations in the GI–hepatic system and its link to the responses of other organs.

## Conclusions and Future Strategy

The consequences of ethanol-induced epigenetic alterations can be positive or negative, depending on the type and duration of the epigenetic changes. Furthermore, the epigenetic responses to ethanol and its metabolites (e.g., acetate) also can differ with a variety of consequences. This diversity remains to be examined thoroughly. Additionally, modifications in DNA and histones located in specific nucleosomes or chromatin domains may differ in their transcriptional effects on various genes, consequently exhibiting varying effects. Alterations in the expression levels of a plethora of miRNAs will add another level of regulatory control over these responses. Finally, it is fair to assume that the diverse epigenetic pathways cross-influence each other, leading to a highly complex regulatory network. The consequences of these epigenetic alterations in the GI tract and liver likely have a systemic impact, influencing other organs and their functions as well, although these interactions are as yet relatively unexplored. Thus, many questions remain that need to be addressed by future research into this area.

## Figures and Tables

**Table 1:** Epigenetic Parameters Altered by Ethanol in the Liver and Gastrointestinal System

**Component**	**Molecular Alterations/Entity**	**Possible Effect On**
**DNA**	DNA methylation via DNA methyl transferase (DNMT) enzymes DNMT1, DNMT3a, and DNMT3b	Alcohol dehydrogenase (ADH), genes for folate metabolism
**Histone**	**Type of modification**	
	Acetylation	ADH, LSD
	Methylation	LSD
	Phosphorylation	C-jun, plasminogen activatory inhibitor 1 (PAI-1)
	**Modifying enzymes**	
	Histone acetyl transferases (HATs)	
	GCN5	
	p300	
	MOZ	
	Histone deacetylases (HDACs)	
	HDAC 1,3,5,6,7,9,10,11	
	SIRT-1	
**micro-RNA**	**Upregulation**	
	miR 03,20,21,29A,34a,101,103	
	miR107, 122, 132,148, 152, 155	
	miR 212, 217, 349, 705, 1224	Lipogenesis
	miR 1256	
	**Downregulation**	Immune response
	miR 19b, 135, 182, 183, 200b	
	miR 199a-3P	
